# Role of Coxsackievirus B3-Induced Immune Responses in the Transition from Myocarditis to Dilated Cardiomyopathy and Heart Failure

**DOI:** 10.3390/ijms24097717

**Published:** 2023-04-23

**Authors:** Fione Yip, Brian Lai, Decheng Yang

**Affiliations:** 1 Department of Pathology and Laboratory Medicine, University of British Columbia, Vancouver, BC V6T 2B5, Canada; fionetsz@student.ubc.ca; 2 The Centre for Heart Lung Innovation, St. Paul’s Hospital, Vancouver, BC V6Z 1Y6, Canada; laibrian@student.ubc.ca

**Keywords:** coxsackievirus B3 (CVB3), myocarditis, dilated cardiomyopathy (DCM), metabolic remodeling, immune response, differentially expressed genes, cytokines, heart failure

## Abstract

Dilated cardiomyopathy (DCM) is a cardiac disease marked by the stretching and thinning of the heart muscle and impaired left ventricular contractile function. While most patients do not develop significant cardiac diseases from myocarditis, disparate immune responses can affect pathological outcomes, including DCM progression. These altered immune responses, which may be caused by genetic variance, can prolong cytotoxicity, induce direct cleavage of host protein, or encourage atypical wound healing responses that result in tissue scarring and impaired mechanical and electrical heart function. However, it is unclear which alterations within host immune profiles are crucial to dictating the outcomes of myocarditis. Coxsackievirus B3 (CVB3) is a well-studied virus that has been identified as a causal agent of myocarditis in various models, along with other viruses such as adenovirus, parvovirus B19, and SARS-CoV-2. This paper takes CVB3 as a pathogenic example to review the recent advances in understanding virus-induced immune responses and differential gene expression that regulates iron, lipid, and glucose metabolic remodeling, the severity of cardiac tissue damage, and the development of DCM and heart failure.

## 1. Introduction

Myocarditis is an inflammatory disease of the heart muscle commonly caused by viruses including coxsackievirus B3 (CVB3), SARS-CoV-2, parvovirus B19, influenza viruses, adenoviruses, and enteroviruses; extensive studies have been executed using CVB3 infection models [1,2,3,4]. The prognosis of myocarditis is diverse; most patients make a recovery, while up to 20% develop chronic myocarditis [5]. Dilated cardiomyopathy (DCM) is a consequence of long-term myocarditis, a severe heart disease characterized by heart enlargement, ventricle chamber dilation, and systolic dysfunction [6,7]. DCM is usually presented with progressive dyspnea, ankle swelling, arrhythmia, thromboembolism, and sudden cardiac death; it has a 5-year survival rate of approximately 50% when left untreated [8]. DCM can also be caused by drugs, toxins, genetics, and metabolic and endocrine disturbances [9]. A high prevalence of viral genomic RNA has been demonstrated in the heart tissues of patients with DCM, suggesting that viral myocarditis plays a significant role in causing DCM [10,11]. However, the molecular pathways that lead to the transition from viral myocarditis to DCM and heart failure are complex. Previous research revealed that viral pathogenesis of myocarditis is attributed to direct damage of cardiomyocytes by viral proteases and immune- and autoimmune-mediated cardiac injury. The cleavage of host immune proteins by proteases is consequential, causing several downstream effects that further progress cardiomyopathy. This review will discuss processes during CVB3 infection that damage host cells, including changes in iron homeostasis and energy metabolism, impairment of mitochondrial function, and alterations in immune profiles that promote tissue damage. More specifically, it will focus on the relevance of the immune response in these processes during the transition of CVB3-induced myocarditis to DCM, as well as recent advances in understanding these mechanisms. Unless otherwise stated, the findings reviewed were obtained from myocarditis mouse models infected with CVB3.

## 2. Immune-Associated Pathogenicity of Coxsackievirus B3 (CVB3)

### 2.1. CVB3 Proteases 2A and 3C Cleave Proteins Involved in Immune Responses

**Figure 1:**
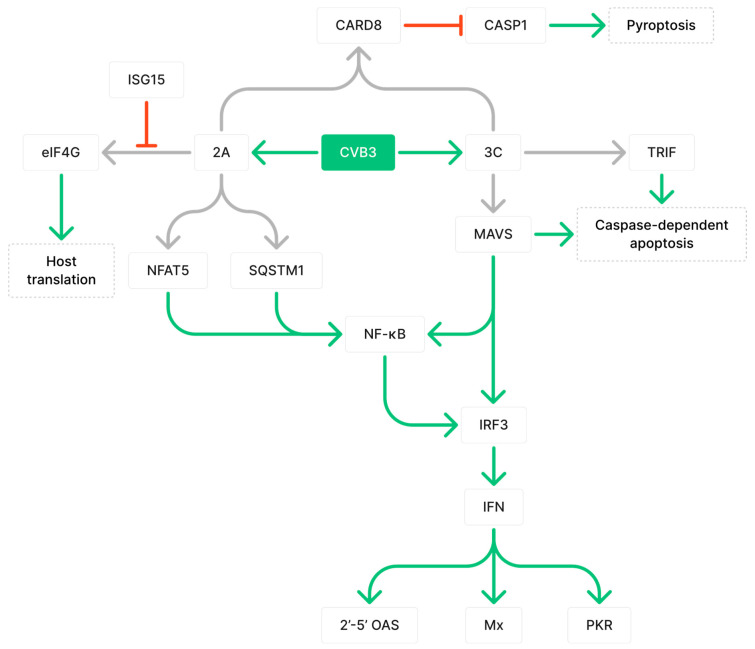
Coxsackievirus B3 (CVB3) protease-mediated alteration of host immune responses by cleaving target proteins. Grey arrows indicate cleavage, green arrows indicate upregulation, and red arrows indicate downregulation. Figure includes 3C protease cleaves mitochondrial antiviral-signaling protein (MAVS) and TRIF, which are important for downstream antiviral responses such as the activation of NF-κB, 2′-5′ OAS, PKR, and Mx, and caspase-dependent apoptosis. As shown, 2A protease disrupts NF-κB activation by cleaving NFAT5 and SQSTM1. Cleavage of eIF4G by 2A to disrupt translation can be inhibited by ISG15. Both 2A and 3C are required for cleavage of CARD8, which normally functions to inhibit activation of CASP1 and pyroptosis.

Infectious pathogens can directly damage host cells by introducing foreign proteins that are toxic to host cells. In CVB3 infections, viral proteases 2A and 3C can impair cellular functions by cleaving an array of host proteins (Figure 1). Host eukaryotic translation initiation factor 4 gamma (eIF4G) is a well-studied target of CVB3 proteases. Cleavage of eIF4G by CVB3 2A halts host protein synthesis and induces cell apoptosis [12]. Other protease substrates include intercalated disk structural proteins desmocollin-2 (DSC2) and desmoglein-2 (DSG2), which are important for binding and signal transmission between myocardial cells to maintain the integrity of the myocardium; TRAF6-binding protein (T6BP), which is involved in clearing damaged mitochondria; and death-associated protein 5 (DAP5), a translation initiation factor that can enhance host cell apoptosis when truncated [13,14,15,16]. The cleavage of numerous host proteins by CVB3 proteases can impair cellular functions to expedite cardiac cell necrosis and, thus, impair cardiac structure and function. This paper will focus on proteins that are associated with the immune response during CVB3 pathogenesis and disease transition. Thus, only select proteins cleaved by CVB3 proteases will be reviewed out of an extensive list.

3C viral proteases can hinder host immune defense by cleaving mitochondrial antiviral-signaling protein (MAVS) and TIR-domain-containing adapter-inducing interferon-β (TRIF) [17]. MAVS is the adaptor molecule downstream of both melanoma differentiation-associated protein 5 (MDA5) and retinoic acid-inducible gene I (RIG-I) viral DNA detectors, which mediate the activation of nuclear factor κB (NF-κB) and interferon regulatory factor 3 (IRF3) [17]. The cleavage of MAVS has been shown to diminish type 1 interferon (IFN) signaling and is important for the expression of effector proteins 2′-5′ OAS, Mx, and PKR, which collectively help degrade and inhibit viral RNA translation [17,18]. Furthermore, MAVS and TRIF are implicated in caspase-dependent apoptosis, which eliminates infected cells to suppress viral replication and prevent viral dissemination [17,19,20,21]. By cleaving MAVS and TRIF, the immune response can be dampened and cell apoptosis can be reduced, leading to prolonged myocarditis and enhanced viral propagation [17]. 2A viral proteases can cleave nuclear factor of activated T cells 5 (NFAT5), a transcription factor in the NF-κB signaling pathway for the transcription of proteins involved in immune responses against cellular stress, including inducible nitric oxide synthase (iNOS) [13,22]. Furthermore, 2A can cleave sequestosome 1 (SQSTM1), an adaptor protein that loses its ability to activate the NF-κB pathway upon cleavage, thus diverting resources from host protein synthesis to viral protein synthesis [16,23]. To counteract this mechanism, the host immune system can induce the expression of IFN-stimulated gene 15 (ISG15), which binds to 2A and inhibits its ability to cleave eIF4G [24]. ISG15-deficient mice were shown to have significantly increased CVB3 virus titers at 8 days post-infection (dpi), greater areas of inflammation that were predominantly composed of macrophages, persistent viral RNA at 28 dpi, and fibrotic tissue development [24]. Proteases 2A and 3C are both required for the cleavage of caspase recruitment domain protein 8 (CARD8) inflammasome, which results in inflammasome activation, caspase 1 (CASP1) activation, and CARD8-driven pyroptosis [25]. Thus, CVB3 proteases cleave an array of host proteins to delay and prolong infection and impair normal host functions.

### 2.2. CVB3 Indirectly Impairs Cardiac Function by Inducing Inflammation That Results in Cardiomyocyte Necrosis and Fibrosis

Infectious pathogens can indirectly damage the myocardium by triggering and sustaining immune responses. Viral double-stranded RNA, including replication intermediates of single-stranded viral genomes, can be detected by Toll-like receptor 3 (TLR3) that is localized to cell surfaces and endosomes [26,27,28]. TLR7 and TLR8 can detect single-stranded RNA, such as the CVB3 genome, whereas TLR9 can recognize unmethylated cytosine–phosphate–guanosine (CpG) DNA, which is abundant in viral genomes [29,30]. After being activated by viral components, TLRs form dimers that recruit myeloid differentiation primary response 88 (MyD88)/TRIF-related adaptor molecule (TRAM) adaptor proteins [31]. These proteins activate transcription factors specific to each pathway, including activator protein 1 (AP-1), NF-κB, and IRF3 [31]. Cytoplasmic helicases RIG-I and MDA5 can recognize distinct viruses and activate type 1 IFN production [32]. For instance, MDA5 activation induces IFN-α production, TLR3 activation induces interleukin 12 (IL-12) production, and the activation of both MDA5 and TLR3 induces IL-6 production [32]. Type 1 IFNs activate the production of ISGs to promote antimicrobial states which limit infection and recruit innate immune cells [33]. Various cytokines are important for cellular communication and viral clearance; however, they can also cause cytokine storms and significant cell stress. Consequently, studying the effective time points and interactions between cytokines will provide valuable information for understanding viral pathogenesis.

Collagen deposition and modification of the extracellular matrix (ECM) are compensatory repair mechanisms used to stabilize the site of injury. However, an imbalance of collagen synthesis and degradation by myofibroblasts can result in cardiac fibrosis, a condition characterized by excessive collagen deposition, which forms permanent scar tissue that disrupts cardiac function and decreases contractile efficiency [34]. These alterations contribute to cardiac remodeling, a process of interstitial changes that includes changes in the size, structure, stiffness, and functioning of the heart, all of which contribute to heart failure [35]. Altogether, the fibrotic response and the immune response work together to repair tissue damage caused by viral infection and inflammation. Significant fibrosis can be detected in mice by day 21 of CVB3 infection [36]. Granulocytes, monocytes, macrophages, and dendritic cells (DCs) contribute to cardiac fibrosis by producing profibrotic cytokines tumor necrosis factor alpha (TNF-α) and transforming growth factor beta (TGF-β) [36,37]. The differentiation of fibrocytes, which produce type 1 collagen, is largely dependent on CD4^+^ T cells and the conditions that activate these T cells [38]. The supporting factors supplemented by CD4^+^ T cells play important roles in the differentiation of monocytes into fibrocytes [38]. The presence of IL-2, IL-4, TNF, or IFN-γ due to polyclonal T-cell activation can inhibit the differentiation of murine monocytes into fibrocytes [38]. Specific combinations of those cytokines resulted in nearly complete suppression of fibrocyte differentiation and collagen deposition [38]. Since numerous immune responses are associated with fibrosis, additional fibrotic cytokines will be reviewed in Section 4.

## 3. Inflammation-Associated Metabolic Remodeling during CVB3-Induced Myocarditis

### 3.1. Impaired Mitochondrial Functions and Ferroptosis Caused by CVB3 Infection and Altered Iron Metabolism

Iron is an essential mineral for energy metabolism and is a component of iron–sulfur cofactors found in several complexes of the mitochondrial respiratory chain [39]. Iron deficiency is a prevalent characteristic of cardiac disorders, such as dilation, left ventricular (LV) hypertrophy, and fibrosis; studies have demonstrated that iron deficiency can reduce cardiomyocyte contractility and relaxation, leading to complications in cardiac function [40,41]. A study on myocarditis and iron homeostasis found that serum from myocarditis patients showed decreased iron levels, along with increased levels of ferritin, an iron-storing protein [42]. This indicates an alteration in iron homeostasis and an increased iron demand during myocarditis [42]. Further, cardiomyocytes treated with serum from myocarditis patient showed elevated levels of transferrin receptor 1 (TFR1), a receptor for iron import that is upregulated in response to low intracellular iron levels [42,43]. TFR1 expression was positively correlated with IL-6 and C-reactive inflammatory protein (CRP) levels from patient sera, suggesting a relationship between inflammation and iron demand [42]. Iron metabolism also showed differences between cardiomyopathy etiologies. Virus-positive cardiomyopathy presented with greater iron demand compared to virus-negative cardiomyopathy, indicating the potential influence of inflammatory profiles on the progression of cardiomyopathy [44]. These findings suggest that inflammation can disrupt iron metabolism and cause iron deficiency, which can impair mitochondrial function and cardiac function.

**Figure 2:**
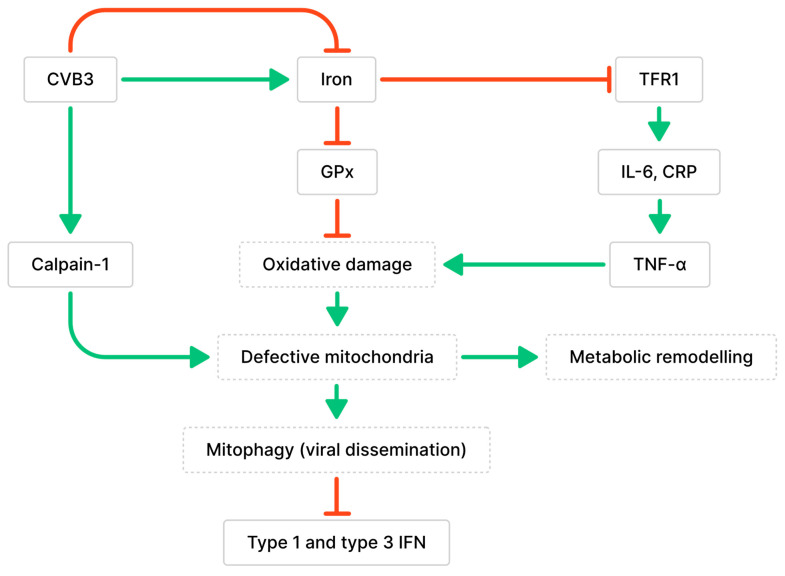
Relationship between CVB3 infection, iron metabolism, and mitochondria dysfunction. Green arrows indicate associated stimulative effects, while red arrows indicate associated inhibitory effects. CVB3 has been associated with altered iron homeostasis (both up- and down-regulated). Altered iron homeostasis can impair peroxidase activity or increase inflammation, which contributes to defective mitochondria, metabolic remodeling, and altered immune responses.

Iron overload has also been associated with cardiomyopathy. Ferroptosis is a form of cell death caused by excessive amounts of iron, which interferes with the antioxidative functions of glutathione peroxidase (GPx) (Figure 2) [45]. This results in iron-dependent lipid peroxidation and leads to oxidative cell death [45]. During CVB3 infection, the myocardium showed a significant increase in ferrous iron levels, which can lead to the generation of hydroxyl radicals through Fenton reactions and damage biomolecules [46,47]. This was observed along with changes to mitochondrial morphology, such as defective cristae, condensed mitochondrial membranes, and the accumulation of malondialdehyde (MDA), which demonstrates increased lipid peroxidation [46]. Iron accumulation and increased viral RNA transcripts were detected in cardiomyocytes as early as 6 dpi [48]. Iron deposits remained in damaged cardiomyocytes 2 months after CVB3 infection, even after inflammation was diminished [48]. These findings demonstrate that both increases and decreases in iron levels occur during myocarditis and are destructive. However, the determinant of iron levels during infection—whether biological differences, diet, or pathology—is unclear.

The accumulation of calpain-1, the main calpain involved in CVB3-induced myocarditis, was observed in the mitochondria during CVB3 infection [49]. Calpains are proteases activated by intracellular Ca^2+^ that are involved in cell necrosis and have been implicated in the development of cardiac fibrosis and dysfunction following long-term CVB3 infection [49]. Calpains initiate pyroptosis, a form of necrotic and inflammatory programmed cell death, by activating the NLR family pyrin domain-containing 3 (NLRP3) inflammasome [49]. Calpain cleaves calcineurin A into its active form, which dephosphorylates dynamin-related protein 1 (Drp-1) and promotes the translocation of Drp-1 from the cytoplasm to the outer mitochondrial membrane [50]. The translocation of Drp-1 activates mitochondrial fission and results in the release of cytochrome c, indicating the activation of apoptosis [50]. CVB3 infection may also contribute to cardiomyocyte apoptosis by increasing Drp-1 expression [50]. Additionally, calpain-1 contributes to the loss of mitochondrial membrane potential (MMP), alterations to mitochondrial structure, and cleavage of ATP synthase-α (ATP5A1) involved in energy production, leading to a decrease in ATP-linked respiration during CVB3 infection [49]. The loss of cardiomyocyte MMP was positively correlated with the amount of CVB3 virions, suggesting that CVB3 infection promotes mitochondrial membrane depolarization in cardiomyocytes [51]. Mitochondria were found to co-localize with lysosomes in CVB3-infected cardiomyocytes and were degraded by PTEN-induced putative kinase protein 1 (PINK1)/Parkin-mediated mitophagy; this finding is consistent with previous observations of mitophagy induced by the loss of MMP [51]. Mitochondrial fragmentation during CVB3 infection resulted in the formation of mitophagosomes that can aid in viral dissemination [51,52]. Additionally, the degradation of RIG-I and MDA5 was observed along with impaired recruitment of MAVS due to Parkin-mediated K48-linked polyubiquitination [51,53]. Since MAVS is essential for mediating interactions with the innate antiviral response kinase TANK-binding kinase 1 (TBK1) and IRF3, Parkin-mediated mitophagy can also result in the suppression of type 1 and 3 IFN production [51,53].

### 3.2. Impaired Lipid and Glucose Metabolism Mediated by CVB3-Induced Inflammation

Anomalies in energy metabolism caused by inflammation can compromise cell and organ function [54]. During acute CVB3-induced myocarditis, a transcriptomic analysis of patients suggests that genes belonging to several metabolic pathways, including fatty acid β-oxidation, the TCA cycle, and the electron transport chain (ETC), are downregulated [54]. ETC complex protein levels and cytochrome c oxidase enzyme activity were decreased, indicating anomalies in mitochondrial oxidative phosphorylation [54]. The infected hearts also expressed decreased levels of mitochondrial transcription factor A (TFAM), which is a mitochondrial biogenesis regulator that enables mitochondrial DNA transcription and maintenance [54,55,56,57]. Since the heart is an energy-demanding organ, impairments in mitochondrial function can significantly contribute to the cardiac diseases [49]. Cardiac metabolic remodeling was supported by the evidence of decreased ATP, ADP, AMP, NAD, and cardiolipin, and an increase in UDP-GlcNAc and arachidonic acid in CVB3-myocarditis hearts [54]. UDP-GlcNAc modifies proteins by O-GlcNAcylation, which can alter protein function and key cellular processes [58]. Cardiolipin is a phospholipid found mainly in the inner mitochondrial membrane; modified species of cardiolipin from remodeling can cause oxidative stress and mitochondrial dysfunction [59]. The oxygenation of arachidonic acid through cyclooxygenases or lipoxygenases leads to the production of prostaglandins and leukotrienes, respectively, which are known mediators of inflammation [60]. Thus, these cardiac metabolites are potential contributors to cardiac remodeling following CVB3 infection.

Adiponectin (APN) is a cytokine secreted by adipose tissues and a regulator of homeostatic pathways such as lipid and glucose metabolism in distant tissues [61]. However, emerging studies have demonstrated higher levels of APN in the coronary sinus compared to the aortic root, suggesting that APN is also synthesized within the heart and may contribute to the development of cardiovascular diseases [62]. APN interrupts TLR3 signaling in cardiac and immune cells by inhibiting the expression of CD14, which is the co-receptor crucial for TLR signaling [63]. In turn, T cell responses are reduced during the sub-acute phase of myocarditis along with diminished viral clearance [63]. In APN knockout mice, the upregulations of IFN-β, IFN-γ, TNF-α, IL-1β, IL-6, and IL-12 were restored to levels comparable to those observed in non-infected mice [63]. During myocarditis, IL-12 signaling increases IL-1β and IL-18, while IL-12 deficiency decreases inflammation and viral replication during myocarditis [64]. In mice, TLR4 deficiency has been shown to decrease levels of IL-1β and IL-18, as well as viral replication and myocarditis [65]. This suggests that TLR4 may share a downstream pathway with IL-12, given the similarities in the pathogenicity between these phenotypes [65]. IL-13 also regulates IL-1β and IL-18 levels by decreasing CASP1 activation, which downregulates the conversion of inactive precursors to active forms of IL-1β and IL-18 [66]. Upregulated T cell proliferation and expression of the pro-inflammatory cytokines IL-1β, IL-18, IFN-γ, TGF-B1, and IL-4 can suppress regulatory T cells, leading to increased anti-cardiac myosin autoantibodies, inflammation, and cardiac fibrosis [66]. APN deficiency accelerates viral clearance while reducing inflammation, necrotic lesions, and the release of troponin I, ultimately maintaining left ventricular function [63].

CVB3 may indirectly cause a reduction in energy metabolism during myocarditis. Notably, oxidative phosphorylation or fatty acid β-oxidation gene expression, including ETC complexes I and III and very long-chain acyl-CoA dehydrogenase (VLCAD), remain unchanged during CVB3 infections lasting up to 72 h [54]. Instead, CVB3 infection increases the expression of cardiac TNF-α, an activator of the NF-κB immunoregulatory pathway, and NF-κB pathway proteins, including NF-κB inhibitor alpha (IκBα) and transcription factor p65 [54]. TNF-α levels were inversely correlated with the expression of VLCAD, ETC complexes I, II and III, and peroxisome proliferator-activated receptor (PPAR) gamma 1 alpha (PGC-1α) [54]. Together, the transcriptomic analyses suggest that CVB3 infection induces the release of cytokines that contribute to energy metabolism anomalies. Increased anaerobic glycolysis is evidenced by increased expressions of glucose transporter-1 (Glut1), lactate dehydrogenase-1 (LDH-1), lactate transporter monocarboxylate transporter-4 (MCT-4), and other glycolytic enzymes [54]. The increase in anaerobic glycolysis suggests that energy production is diverted from the normal aerobic pathway in the mitochondria. This can be a result of insufficient oxygen supply, damage to mitochondrial respiratory chain proteins, or increased energy demand, which can occur when mitochondrial functions are impaired.

The transcripts of fatty acid metabolism regulators PGC-1α, PGC-1β, and PPAR-α were found to be decreased in viral myocarditis hearts [54,57]. PPARs are anti-inflammatory nuclear receptors that can be expressed in immune cells [67]. They function as a transcription factor and can regulate the activity of other transcription factors, such as the inhibition of NF-κB [67]. The activation of PPAR-α inhibits Th17 cell differentiation by suppressing IL-17, IL-6, TGF-B, p-STAT3, and ROR-γt expression, which are critical for Th17 differentiation [68,69]. PPAR-α and its coactivators stimulate lipid catabolism by increasing fatty acid uptake, fatty acid oxidation, and lipoprotein assembly; they are highly expressed in organs that depend largely on oxidative metabolism for energy [70,71,72]. The deficiency of PGC-1α and PGC-1β has been shown to accelerate heart failure, leading to oxidative stress, decreased cardiac deficiency, and reduced glucose metabolism [54,73,74]. These observations coincided with decreased ejection fraction and fractional shortening. Moreover, these findings suggest that metabolic remodeling begins as early as acute myocarditis, it occurs before structural remodeling, and it contributes to cardiomyocyte dysfunction by driving cell death [54].

## 4. Immune-Associated Cells and Genes That Influence Cardiac Remodeling and Dilated Cardiomyopathy (DCM) Development

In response to infection, innate immune cells are recruited to tissues to phagocytose pathogens and mediate pro-inflammatory and antiviral responses [75]. Adaptive immune cells include T cells and B cells with different subsets, cytokine profiles, and memory and effector functions [76,77]. Fibroblasts are involved in injury repair and produce connective tissue such as collagen to provide the structural framework for organs and tissue [78]. This section will review various genes expressed by immune cells and fibroblasts that contribute to the duration and severity of myocarditis and development of DCM (Table 1).

### 4.1. Monocytes

Monocytes are recruited to infected tissues and can differentiate into macrophages or DCs to phagocytose pathogens and mediate pro-inflammatory and antiviral responses. Heart tissue biopsies from patients with myocarditis revealed an increase in the number of monocytes that express CCR2 [90]. CCR2 facilitates monocyte egression from the bone marrow into the blood and their subsequent migration into inflammatory sites [91]. The silencing of CCR2 in CVB3-infected mice significantly was shown to reduce the accumulation of lymphocyte antigen 6 complex (Ly6C) high monocytes in cardiac tissue during acute myocarditis [90]. The presence of Ly6C^high^ monocytes is crucial for CD8^+^ T cell responses, as demonstrated by a shift in immune responses when these monocytes are absent; this shift is characterized by increased bone marrow neutrophil expansion and preserved cardiac function, as measured by left ventricular ejection fraction [36,90]. Delayed silencing of CCR2 improved late-stage disease outcomes for myocarditis in mice at 60 dpi by reducing inflammation and fibrosis [90]. Monocyte recruitment is also mediated by stabilin-1, a receptor expressed on myeloid cells that facilitates adhesion with extracellular fibronectin [88]. Stabilin-1 deficiency led to a significant reduction in the recruitment of both pro-inflammatory (Ly6C^high^) and anti-inflammatory (Ly6C^low^) monocytes [88,89]. Since stabilin-1 regulates monocyte differentiation into regulatory macrophages, a deficiency in stabilin-1 drastically reduces the number of cardiac anti-inflammatory macrophages, which are crucial for suppressing T cell responses [88]. Thus, stabilin-1-KO mice showed increased pro-inflammatory responses, cardiac necrosis, and mortality during myocarditis [88].

Following CVB3 infection, monocytes displayed differentially expressed genes in three different clusters. Monocyte cluster A had anti-inflammatory and tissue repair functions with upregulated levels of M2 macrophage markers [113]. This cluster helped with myocardial repair and cardiomyocyte cell survival by expressing increased transcript levels of transforming growth factor-beta-induced protein (TGFBI) and S100 calcium binding protein A4 (S100A4) [113]. Monocyte cluster B regulated pathways involved in cardiac muscle contraction and hypoxia by expressing upregulated levels of CCL24, CCL2, galectin 1 (Gal-1), and WAP four-disulfide core domain 17 (WFDC17) transcripts [113]. Monocyte cluster C also had an anti-inflammatory phenotype, expressing increased levels of the inhibitory Fc receptor Fc gamma receptor IIb (FCGR2B), IL-1β suppressor membrane-spanning 4-domains (MS4A6D), and arginine metabolism activator glycine amidinotransferase (GATM) [113]. Thus, the different populations of monocytes that infiltrate the heart at 21 dpi have increased expressions of genes, contributing to an anti-inflammatory state [113].

### 4.2. Macrophages

Interleukin-1 receptor-associated kinase 4 (IRAK4) is a signal transducer that mediates the translocation of NF-κB after TLR or T cell receptor (TCR) activation [100]. While IRAK4 supports the production of pro-inflammatory cytokines, it also promotes CVB3 infection by blocking the migration of early protective macrophages and the production of IFNs [100]. In mice with myocarditis, IRAK4 deficiency improves heart functions and partially reverses the decrease in fractional shortening caused by CVB3 infection [100]. Since IRAK4 negatively regulates Stat5, the major transcription factor of CCR5, it limits CCR5-dependent migration of monocytes to the heart during myocarditis [100]. Interestingly, IRAK4 only modulates CCR5 expression in monocytes and macrophages; it does not modulate T cells which also express CCR5 [100]. The absence of CCL5 and CCR5 in mice led to higher mortality rates and enhanced viral proliferation, which demonstrates that infiltrating CD11b^+^F4/80^+^ monocytes and macrophages at 2 dpi play a crucial role for containing the infection [100]. IRAK4 also contributed to the dimerization of IRFs, which are transcription factors involved in the synthesis of type 1 IFNs [30,114]. IRF5/IRF7 heterodimers inhibit the transcription of IFN-α, while IRF5 homodimers can activate IFN-α transcription [114]. IRAK4 has been shown to favor IRF5/IRF7 heterodimerization in macrophages, thus inhibiting type 1 IFN-mediated antiviral responses [100]. Altogether, IRAK4 exacerbates myocarditis by decreasing the early influx of protective monocytes and macrophages to the heart, as well as inhibiting type 1 IFN production, which ultimately benefits viral replication. Type 1 IFN signaling is also inhibited through the STAT3 signaling pathway by TRIM18, an E3 ubiquitin ligase that mediates the ubiquitination of protein phosphatase 1A (PPM1A) [112]. TRIM18 recruits PPM1A to dephosphorylate and inactivate TBK1 [112]. Thus, TBK1 can no longer interact with its upstream adaptors MAVS and STING and attenuates type 1 IFN signaling [112]. The knockdown of TRIM18 considerably increases type 1 IFN signaling and has been shown to increase IFN-α and IFN-β production by macrophages during viral myocarditis [112].

Substantial amounts of infiltrating macrophages can be observed in the myocardium during CVB3 infection [97]. These macrophages secrete IL-1β, which is a cytokine implicated in the transition from acute myocarditis to inflammatory cardiomyopathy [97,115,116]. During acute myocarditis, IL-1β elevates the expression of matricellular proteins tenascin C (TN-C) and osteopontin (OPN) [97]. TN-C is an activator of pro-fibrotic responses in fibroblasts and increases the rate of fibroblast migration across injured tissues and collagen deposition [107]. Additionally, OPN induces fibrotic tissue development and cardiac remodeling by promoting the formation of insoluble collagen, which contributes to LV stiffness and the development of systolic dysfunction [101]. IL-1β also decreases cardiac lymphatic muscle cell contractility in a dose-dependent manner [98]. This may hinder the clearance of inflammatory cells and cytokines and prolong tissue damage, leading to cardiomyopathy [98]. While the impediment of viral clearance is a concern when using anti-inflammatory therapies, the neutralization of IL-1β can prevent fibrosis without increasing viral load in the heart and presents a potential therapeutic target [97].

Macrophages secrete large amounts of Gal-3 during acute and chronic CVB3-induced myocarditis to mediate both the migration of macrophages to fibroblasts and the transformation of fibroblasts into matrix-secreting myofibroblasts [93,117,118]. Although the inhibition of Gal-3 may not prevent viral clearance, it reduces acute inflammation, collagen secretion, and the expression of A- and B-type natriuretic peptide (ANP and BNP) mRNA, which are markers of cardiac dysfunction [93]. The absence of macrophages leads to a reduction in Gal-3^+^ cells in the heart normally observed during CVB3 infection, a decline in myofibroblast activation, and fewer collagen deposits [93]. This resulted in viral clearance by 30 dpi and the attenuation of acute myocarditis [93]. These findings demonstrate that inflammatory macrophages encourage the development of fibrosis following myocarditis by secreting Gal-3. Cardiac-infiltrating macrophages express upregulated levels of dipeptidase 2 (Dpep2) during myocarditis [79]. Dpep2 protects mice from inflammatory macrophages in the acute phase by suppressing the activation of the NF-κB pathway to limit the expression of TNF-α, IL-6, and monocyte chemoattractant protein 1 (MCP-1) in response to infection [79].

### 4.3. Neutrophils

The increase in neutrophils circulating in both the blood and heart can be detected within 24 h of infection [36]. On day 3, neutrophils were found to be more abundant in the heart compared to macrophages and monocytes, making them the predominant immune cell present [36]. Gene ontology analyses identified the upregulation of pathways involved with IL-17, NF-κB, TNF, IL-1β and IL-4 signaling, cell death, and viral life cycle regulators in neutrophils during myocarditis [113].

Damage-associated molecular patterns (DAMPs) are immune-activating proteins that are secreted after cell injury or infection and include interleukins, heat-shock proteins, high-mobility group box 1 (HMGB1), and S100 proteins [119,120]. S100A8 and S100A9 are mainly expressed and secreted by neutrophils and monocytes upon cellular activation [121,122,123]. They are implicated in inflammation, including the release of IL-6, IL-8, CCL2, CCL20, and CXCL10 in tendinopathy and the activation of neutrophils in COVID-19 patients [124,125]. The activation of NADPH oxidase 1 (Nox1) to produce reactive oxygen species (ROS) can be initiated by the binding of S100A9 to the receptor for advanced glycation end products (RAGE) [104]. Monocyte-derived macrophages overexpressing S100A8 and S100A9 show increased ROS production and IL-10 mRNA expression [123]. The increase in IL-10 may be a defensive mechanism since controlled macrophage activation is influenced by the levels of IFN-γ and IL-10 [126]. IL-10 deficiency is associated with the uncontrolled production of iNOS, which catalyzes the production of nitric oxide (NO) and contributes to ongoing tissue damage [126]. S100A9 deficiency in CVB3-infected mice leads to a significant decrease in RAGE-dependent ROS production in the left ventricle, which can be reversed with S100A9 supplementation [104]. Moreover, S100A9 deficiency is associated with reduced numbers of neutrophils and monocytes in the left ventricle, as well as decreased CXCL2 secretion by monocytes and neutrophils at inflammation sites [104]. This finding suggests that S100A9 promotes the accumulation of monocytes and neutrophils in the left ventricle during myocarditis.

S100A9 may also contribute to tissue damage by increasing granulocyte activity or causing mitochondrial dysfunction. A recent study has demonstrated that S100A8 and S100A9 are released by neutrophils recruited to infarcts in the heart [127]. S100 proteins can bind to TLR4s on naïve neutrophils to stimulate IL-1β secretion and induce granulocyte production [127]. S100A8 and S100A9 have been demonstrated to cause mitochondrial respiratory dysfunction by TLR4 signaling that inhibits mitochondrial complex 1, resulting in the death of cardiomyocytes in mice with ischemic/reperfusion injury [105]. After CVB3 infection, the absence of S100A9 was associated with improved global heart function, including ejection fraction, cardiac output, stroke work function, and systolic and diastolic functions [104]. Endomyocardial biopsies from patients with CVB3-positive myocarditis expressed a significant increase in S100A8 and S100A9 compared to healthy controls [104]. These levels were reduced once the virus was cleared, suggesting that S100A8 and S100A9 expressions are induced by CVB3 infection [104]. CVB3 infection notably results in increased levels of S100A8 and S100A9 in the left ventricles of mice [104]. However, S100A8 and S100A9 are not differentially expressed in CVB3-negative DCM patients, further suggesting that their expression levels are dependent on the viral load [104]. Interestingly, S100A9 has been associated with higher expression of coxsackievirus and adenovirus receptor (CAR) mRNA and decreased IFN-β in CVB3-infected mice, which can contribute to the increase in CVB3 RNA copy numbers in mice [104]. IFN-β deficiency increases susceptibility to disease by downregulating the expression of ISGs, including 2′-5′ OAS, which degrades both viral and host RNA, and GTPase Mx, which binds to the viral components of various viruses to block their functions [18,128]. The current findings suggest that S100A8 and S100A9 aggravate CVB3-induced myocarditis by increasing the accumulation of myeloid cells that can release tissue-damaging granules, are mainly involved in the earlier stages of disease that leads to impaired heart functions, and do not appear to play a role in DCM when the virus is absent [104].

### 4.4. T Cells

Mice with myocarditis corresponded with significantly higher numbers of CD8^+^ cytotoxic T lymphocytes, Th17 cells, and CD4^+^ T cells that express TCF4, KI-67, and CCR1 [113]. CD4^+^ T cells in mice with myocarditis had enhanced expressions of CCL5, which can induce NF-kB activation; NKG7, which is associated with cytotoxicity; programmed cell death protein 1 (PDCD1), which is associated with T cell exhaustion; and inhibitor of DNA binding 2 (ID2), which mediates Treg plasticity into Th17 cells [113,129,130,131]. CD8^+^ T cells expressed elevated levels of CCL5, S100A6, S100A4, PDCD1, CXCR6, and BCL2A1B transcripts [113]. The immunophenotype of patients with myocarditis and left heart failure was characterized by a Th17 response and decreased levels of Tregs [94]. Since CVB3 VP1 has a similar epitope to cardiac myosin, cardiac myosin exposure after cardiomyocyte damage has been demonstrated to promote heart failure by inducing Th17 immune responses [94,132]. Myocarditis and DCM can be characterized by elevated Th17 pathogenesis and release of the associated cytokines TGF-β1, IL-6, IL-23, and granulocyte-macrophage colony-stimulating factor (GM-CSF), which are correlated with heart failure [94]. While cardiac myosin is normally concealed, exposed fragments of cardiac myosin fragments act as danger signals and are recognized as TLR2 ligands [94]. Monocytes activated by cardiac myosin produce elevated levels of TGF-β1, IL-6, IL-23, and IL-1β [94]. TGF-β1 and IL-1β initiate Th17 differentiation, which can be enhanced by IL-6 [94,95,133,134,135]. IL-23 supports pathogenic Th17 cell activity, such as GM-CSF secretion [94,95,133,134,135]. IL-6 was persistently elevated at 6 and 12 months in non-recovery patients, while IL-17A was associated with fibrosis in DCM patients with class III-IV heart failure [94]. Thus, cardiac damage due to CVB3 infection induces Th17 responses that support the development of chronic myocarditis and heart failure [94].

Both Th1 and Th17 cells infiltrate the heart during myocarditis. However, TH17 responses result in more tissue damage than Th1 cell responses [96,136]. Compared to Th1 cells, a greater number of Th17 cells co-secrete TNF-α and IL-6, which are associated with heart failure [96,136]. IL-10 secretion is also reduced in Th17 cells compared to Th1 cells [96,136]. CD80 and CD86 are costimulatory molecules expressed by antigen-presenting cells; both molecules can interact with CD28 and CTLA-4 receptors on T cells to regulate T cell activation and cytokine production [137]. Both CD80 and CD86 arose from gene duplication and have shared functions; however, only CD80 exacerbates myocarditis [92]. CD80 was found to upregulate ROR-γt expression in heart tissue, leading to increased production of IL-17 by CD4^+^ T cells and promoting Th17 cell differentiation [92,138]. Although CD86 alone does not affect Th17 cell populations, it is involved in the balance of Th17 cells by neutralizing the effects of CD80 [92]. IL-17A, which is largely secreted by Th17 cells, affects the proportions of infiltrating cell populations [96]. It mediates the recruitment of monocytes, eosinophils, and neutrophils to the heart by upregulating several chemokines that direct their migration, including CCL2, CCL3, CCL5, CXCL1, and CXCL10 [96]. Interestingly, while the secretion of IL-17A by Th17 cells may not contribute to the severity of CVB3-induced myocarditis, it plays a role in the development of DCM [96]. The pathogenic effects of IL-17A may depend on the induction of pro-inflammatory cytokines IL-1β, IL-6, and TNF-α, as well as pro-fibrotic cytokine TGF-β1, which were abrogated in the absence of IL-17A [96]. Continual TNF-α secretion can negate improvements in both cardiomyocyte contraction and relaxation mediated by cardiac microvascular endothelial cells (CMECs) [108]. Elevated levels of circulating TNF-α are detected in patients with DCM and have been associated with cachexia [139,140]. The elevated TNF-α levels coincided with inflammatory cell accumulation and fibrogenic responses, contributing to cardiac dysfunction [109]. In mice, TNF-α deficiency attenuates cardiac CASP8 activation and apoptosis in response to pressure overload, demonstrating that TNF-α contributes to cardiac remodeling through caspase-dependent myocyte apoptosis [109]. IL-6 receptor blockade has been shown to attenuate CVB3-induced production of TNF-α [99]. IL-17A mediates left ventricular dilation and is essential for increasing left ventricular end-systolic and end-diastolic diameters [96]. This cytokine also compromises myocardial contractility and contributes to decreased fractional shortening and ejection fraction [96]. IL-17A is associated with fibrotic tissue development and coincides with increasing type 1 and type 3 collagen deposition, gelatinase, matrix metalloproteinase 2 (MMP-2), and MMP-9 biomarkers for severe cardiac remodeling in patients; it is also associated with decreasing tissue inhibitor of metalloproteinases 1 (Timp1) and Timp4 [96,141]. MMPs are enzymes involved in the degradation of ECM components, such as signaling molecules, for release, activation, and inactivation of cytokines, as well as the migration of immune cells [142]. MMP-9 was shown to be necessary for attenuating acute CVB3 infection, presumably by mediating the degradation of IFN-β and IFN-γ [143].

IL-9 is largely secreted by CD8^+^ T cells on day 5 and by CD4^+^ T cells on day 7 of infection [81]. IL-9 inhibits viral replication by reducing CAR expression on cardiomyocytes [81]. The knockout of IL-9 decreases TGF-B secretion while increasing IL-17a secretion [81]. The synergistic enrichment of IL-9, IL-3, IL-4, IL-13, and IL-15 during acute myocarditis can be cardioprotective by significantly increasing the number of cardiac helper T cells, shrinking necrotic lesion sizes, and limiting CVB3 genomes [80]. These synergistic changes can prevent cardiac remodeling and fibrosis, decrease end-diastolic dilatation, and improve systolic function 5 weeks post-infection [80]. IL-4 and IL-13 promote CCL11 secretion by fibroblasts [144]. The overexpression of IL-9 significantly reduces the percentage of Th17 and Th22 cells, which are adversely linked to cardiac remodeling and increased anti-inflammatory Th2 cells at 14 dpi [80].

During myocarditis, signal transducer and activator of transcription 4 (STAT4) promotes the NF-κB pathway by increasing IKBα and p65 phosphorylation in myocardial cells [106]. STAT4-driven NF-κB activity upregulates Th1 inflammatory cytokines IFN-γ and IL-2, while downregulating Th2 anti-inflammatory cytokines IL-6 and IL-10 [106]. The overexpression of STAT4 results in increased focal necrosis, fibrosis, and interstitial hyperplasia, while the silencing of STAT4 and the NF-κB pathway represses the development of myocarditis [106]. STAT4 and Janus kinase 2 (JAK2) phosphorylation can be decreased by progranulin (PGRN), which is a pleiotropic growth factor and adipokine that is constitutively expressed in certain epithelial cells and myeloid- and lymphoid-derived cells [87,145]. PGRN is significantly increased in plasma and cardiac tissues during sub-acute (7 dpi) myocarditis to attenuate CVB3 replication [87]. PGRN is also negatively correlated with cardiac inflammation and the loss of body weight in mice [87]. PGRN directly inhibits Th1 differentiation by decreasing the phosphorylation of JAK2 and STAT4; it also inhibits Th17 cell differentiation by decreasing JAK3 and STAT3 phosphorylation [87]. Mice with PGRN deficiency show significant upregulation in Th1 and Th17 phenotypes and severe myocarditis with necrosis [87]. The pro-inflammatory cytokines IFN-γ, TNF-α, IL-17A, and IL-21 were also found to be upregulated in the absence of PGRN [87]. During CVB3-induced myocarditis, IL-21 promotes the activation of CD8^+^ T cells, which may include autoimmune CD8^+^ T cells that target cardiac tissue [146]. Upregulation of CD1d recognized by γδ T cells to activate autoimmune CD8^+^ T cells requires both an active infection and the presence of TNF-α signaling [110].

### 4.5. B Cells

B cells are important regulators of left ventricular contractility and myocardial leukocytes, including the balance of different myeloid-derived cells and T cell populations [147]. Myocarditis significantly increases B cell activation and antigen presentation, which was observed alongside increased CD69, CD40, CD80, and MHCII expression 7 dpi [148]. B cells can contribute to the development of viral myocarditis through antigen presentation and cytokine production to alter the balance of T cell populations. TNF-α, IL-6, and IL-17 secretion by B cells promoted Th1 and Th17 differentiation during myocarditis, while the downregulation of IL-4 significantly limited Th2 differentiation [148].

B10 cells are regulatory B cells that can limit inflammation by producing IL-10. A significant increase in B10 cells has been observed in the heart at around day 7 of myocarditis [86]. B10 cells can alleviate myocardial damage by decreasing the amount of Th17 infiltrates [86,149]. This population of B cells can downregulate the expression of T-bet and ROR-γt, which are transcription factors for Th1 and Th17 phenotypes, respectively [86,149]. The suppression of Th17 differentiation by B10 cells can be enhanced by prostaglandin E2 (PGE2) treatment [86]. PGE2 is a lipid molecule produced by fibroblasts and macrophages that has anti-inflammatory effects on immune cells, including neutrophils and macrophages [83,84,85]. PGE2 induces the expansion of B10 cells by activating AP-1 and AhR, which are transcription factors that promote the expression of IL-10 [86]. Treatment with PGE2 enhances B10 cell suppression of Th17 differentiation, reduces inflammatory infiltrates, and restrains cardiac hypertrophy during myocarditis [86]. However, B cells in DCM patients were found to secrete elevated levels of TNF-α in comparison to anti-inflammatory cytokines, such as IL-10 [150].

IL-10 is an anti-inflammatory cytokine that inhibits the release of pro-inflammatory cytokines, including TNF-α; thus, it also inhibits myocyte apoptosis induced by TNF-α [82]. TNF-α-induced apoptosis involves the production of ROS by sphingomyelinases through a ceramide-dependent signaling pathway [111]. ROS products can act upon cellular macromolecules and promote lipid degradation and enzyme inactivation [151]. Consequently, cellular respiration and DNA synthesis are inhibited in response to the damage of macromolecules, leading to apoptosis [151]. Nevertheless, superoxides, a major ROS species, can react with NO and cause its depletion from cardiac microvascular endothelial cells, which require NO to favorably enhance cardiomyocyte contraction and relaxation [108]. Patients with DCM and mild congestive heart failure show significant oxidative stress, which can contribute to cardiomyopathic changes [152]. IL-10 interrupts the pro-apoptotic pathway induced by TNF-αby downregulating IKK phosphorylation in manner dependent on the phosphorylation of ERK1/2 MAPK [82].

### 4.6. Fibroblasts

Fibrotic and inflammatory responses work together to promote the healing of damaged tissue caused by pathogen infection and subsequent clearance. Several populations of fibroblasts express elevated levels of C3 and C4b complement proteins; serine protease inhibitor A3N (Serpina3n), which promotes wound healing; and IFN-induced transmembrane protein (Ifitm) during myocarditis [113]. CVB3 triggers the release of TNF-α, IL-1β, IL-6, and CCL2 by fibroblasts; however, it does trigger the release of profibrotic mediators, such as Col I, Col III, or TGF- β [153]. Instead, fibroblasts secrete early stem cell factor (SCF), which triggers mast cells to degranulate and secrete more TNF-α [153]. In return, TNF-α stimulates the production of Col I, Col III, and TGF- β by fibroblasts [153]. The absence of mast cells is associated with reduced fibrosis and improved cardiac dysfunction [153]. Thus, the interaction between mast cells and fibroblasts is crucial for activating fibroblast functions and promoting the generation of fibrotic tissue.

Cardiomyocytes and cardiac fibroblasts were identified as the main producers of IL-6 during myocarditis [99]. IL-6 signaling is highly correlated with ICAM expressions that are important for adhesion and recruitment [99]. When stimulated by IL-6, cardiac fibroblasts produce more IL-6 in a positive feedback loop, which can lead to increased cell necrosis and immune cell infiltration [99]. Mice treated with IL-6 receptor blockade have reduced numbers of infiltrating T cells and macrophages, potentially due to the downregulation of the adhesion molecules that mediate lymphocyte migration [99]. IL-6 receptor blockade during CVB3 infection has been observed to alter titin phosphorylation. Sarcomeres are the basic contractile unit that forms muscle fibers and are composed of different structural protein filaments, including titin. Titin functions like a molecular spring and contains numerous phosphorylation sites that can increase or decrease myocyte stiffness [154]. Phosphorylation of the cardiac-specific N2-B unique sequence (N2-Bus) decreases myocyte stiffness, while phosphorylation of the titin domain rich in proline, glutamate, valine, and lysine (PEVK) increases stiffness [155]. The differing effects are caused by the amino acid charges in the sequence. Phosphorylation of the basic regions in the PEVK domain reduces electrostatic repulsion, which leads to increased intramolecular interactions, thus increasing stiffness [156]. Failing hearts of DCM and hypertrophic cardiomyopathy (HCM) patients displayed N2-Bus hypophosphorylation and PEVK hyperphosphorylation, both of which can contribute to increased myocyte stiffness [156]. IL-6 has been observed to increase myocyte stiffness by inducing titin phosphorylation at the PEVK region [155]. The neutralization of IL-1β markedly reduces the expression of IL-6, which is essential for inducing Th17-mediated pro-inflammatory responses [97,157].

Protease-activated receptors (PARs) are G-protein-coupled receptors that are activated by proteases cleaving the extracellular N-terminus of PAR, which in turn produces a tethered activating ligand [158,159]. Depending on the protease and the resulting ligand sequence produced, activated PARs can couple with different G protein α-subtypes that determine the downstream signaling pathway, such as inflammatory responses, by activating NF-κB [159]. Activated PAR1 in cardiac fibroblasts can stimulate TLR3 to upregulate the phosphorylation of P38 [102]. Phosphorylated P38 promotes the production of CXCL10 [102]. CXCL10 is an ISG that recruits NK cells and CD3^+^ leukocytes to eliminate infected cells and contain the viral infection [160]. However, PAR1 activity may enhance viral replication by reducing autophagic flux [161,162]. The reduction in autophagic flux prevents the degradation of viral RNA, viral proteins, and autophagosomes, leading to an accumulation of autophagosome membranes that can be exploited as scaffolds for virus assembly [162]. Activated PAR2 can directly interact with TLR3 to inhibit TLR3-mediated IFN-β production, which normally stimulates antiviral responses [103]. Thus, the decrease in IFN-β signaling due to PAR2 activation coincides with increased viral replication [103]. PAR2 is also associated with increased expressions of CAR and decay-accelerating factor (DAF), which is the coreceptor of CAR and can increase the rate of viral infection [103]. Mice expressing PAR2 present persisting levels of viral titer, increased CD68^+^ and CD3^+^ cell infiltrates in the heart, more severe tissue damage, significantly higher expressions of TNF-α, IL-6, IL-1β, CCL2, CCL5, and IFN-γ at 8 dpi, and a significant reduction in left ventricular function at 28 dpi compared to PAR2-KO mice [103]. These results suggest that PAR2 impedes the innate immune response and viral clearance by blocking TLR3-signaling, leading to more aggressive adaptive immune responses. These findings were consistent with clinical studies in which patients who expressed higher levels of PAR2 secreted lower levels of IFN-β, had more CD3^+^, CD45^+^, and myocardial infiltrates, and showed impaired heart function measured by left ventricular ejection fraction (LVEF) [103].

## 5. Perspectives

The collection of evidence demonstrates that CVB3-induced myocarditis can impair cardiac function by cleaving host proteins involved in the immune response, including MAVs, TRIF, NFAT5, SQSTM1, and CARD8, to impair host defense mechanisms and promote pyroptosis and apoptosis. CVB3 infection impairs mitochondrial functions through multiple pathways, including altered iron homeostasis and inflammation. Mitophagy following CVB3 infection is inter-related with the immune response, including the inhibition of IFNs. Differential cytokine expression and immune cell phenotypes play an extensive role in determining the outcome of myocarditis. The shift in immune profiles during infection can promote tissue damage, as well as the development of DCM and heart failure. Given that some patients remain asymptomatic while others develop heart failure, biological differences in immune profiles can be significant contributors to disease outcomes. Thus, recently discovered immune-associated genes that mediate the transition from myocarditis to cardiac dysfunction are critical targets for pathology and therapeutic development. As immunostimulant and suppressive therapies have shown inconsistencies due to varying time points of administration and patient variance, it is crucial to understand the complex network of immune cells and cytokines to personalize therapy based on factors including a patient’s genetic profile or the severity of DCM. In genetics research, a focus on identifying new biomarkers may help identify early signs of myocarditis and DCM progression for diagnosis and treatment purposes. Moreover, future research could focus on identifying new genes that lead to DCM and how different mutations in the same gene can impact the severity of DCM. The use of bioinformatics analyses will lead to a better understanding of the relationships between an array of networks that impact the outcomes of myocarditis, including iron, lipid, and glucose metabolism, viral protease activity, cytokine expression, and immune cell responses.

## Figures and Tables

**Table 1:** Proteins that can impact myocarditis and development of cardiomyopathy.

Protein	Myocarditis/ Cardiomyopathy	Function	Effects during CVB3 Infection	Reference
**Dpep2**	Alleviate	Inhibits macrophage NF-κB signaling	Reduced inflammation	[79]
**IL-3, IL-4, IL-9, IL-13, IL-15 (synergy)**	Alleviate	Recruits helper T cells	Reduced cardiomyocyte necrosis Reduced fibrosis Reduced LV anomalies Reduced viral load	[80]
**IL-9**	Alleviate	Reduces cardiomyocyte CAR expression Inhibits Th17 differentiation	Reduced viral load	[80,81]
**IL-10**	Alleviate	Inhibits TNF-α-induced apoptosis	Reduced cardiomyocyte apoptosis	[82]
**PGE2**	Alleviate	Induces anti-inflammatory effects on neutrophils and macrophages Induces B10 expansion Suppresses Th17 differentiation	Reduced LV anomalies Reduced inflammation	[83,84,85,86]
**PGRN**	Alleviate	Inhibits Th1 differentiation Inhibits Th17 differentiation	Reduced inflammation Reduced cardiomyocyte necrosis Reduced viral load	[87]
**PPAR-α**	Alleviate	Inhibits NF-κB Inhibits Th17 differentiation	Reduced inflammation	[67,68]
**Stabilin-1**	Alleviate	Increases monocyte recruitment Promotes monocyte differentiation into regulatory macrophages	Reduced inflammation Reduced cardiomyocyte necrosis Reduced mouse mortality	[88,89]
**APN**	Exacerbate	Decreases TLR signaling Increases cytokine production	Increased inflammation Increased viral load Increased LV anomalies	[63]
**Calpain-1**	Exacerbate	Increases pyroptosis Promotes mitochondrial dysfunction	Increased cardiomyocyte necrosis Increased fibrosis	[49]
**CCR2**	Exacerbate	Increases monocyte recruitment	Increased fibrosis Increased inflammation	[90,91]
**CD80**	Exacerbate	Increases IL-17 production	Increased cardiomyocyte necrosis	[92]
**Gal-3**	Exacerbate	Induces transition from fibroblasts to myofibroblasts Mediates the migration of macrophages towards fibroblasts	Increased fibrosis Increased inflammation	[93]
**GM-CSF**	Exacerbate	Increases Th17 differentiation	Increased inflammation	[94,95]
**IL-17A**	Exacerbate	Increases pro-fibrotic cytokines Increases pro-inflammatory cytokine expression Increases Th17 differentiation Recruits myeloid cells	Increased fibrosis Increased inflammation Increased LV anomalies	[96]
**IL-1β**	Exacerbate	Increases matricellular protein expression Increases Th17 differentiation	Increased fibrosis Increased inflammation Increased LV anomalies	[97,98]
**IL-23**	Exacerbate	Increases GM-CSF secretion	Increased inflammation	[94]
**IL-6**	Exacerbate	Induces titin phosphorylation Increases Th17 differentiation Mediates TNF-α expression Recruits T cells and macrophages	Increased cardiomyocyte necrosis Increased fibrosis Increased LV anomalies Increased viral load	[99]
**IRAK4**	Exacerbate	Inhibits IFN production Inhibits migration of protective macrophages and monocytes	Increased LV anomalies Increased viral load	[100]
**OPN**	Exacerbate	Increases insoluble collagen	Increased fibrosis Increased LV anomalies	[97,101]
**PAR1/2**	Exacerbate	Increases CAR and DAF expression Reduces autophagic flux	Increased inflammation Increased LV anomalies Increased viral load	[102,103]
**S100A9**	Exacerbate	Increases ROS production Inhibits mitochondrial complex 1	Increased inflammation Increased LV anomalies Increased viral load Mitochondrial respiratory dysfunction	[104,105]
**STAT4**	Exacerbate	Upregulates NF-κB pathway	Increased cardiomyocyte necrosis Increased fibrosis Increased inflammation	[106]
**TN-C**	Exacerbate	Increases fibroblast migration	Increased fibrosis	[97,107]
**TNF-α**	Exacerbate	Increases ROS production Activates autoimmune CD8^+^ T cells	Increased cardiomyocyte apoptosis Increased fibrosis Increased inflammation Increased LV anomalies	[108,109,110,111]
**TRIM18**	Exacerbate	Inhibits IFN production	Increased inflammation Increased LV anomalies	[112]

## Data Availability

Not applicable.

## References

[1] Rezkalla S.H., Kloner R.A. (2021). Viral myocarditis: 1917–2020: From the Influenza A to the COVID-19 pandemics. Trends Cardiovasc. Med..

[2] Mele D., Flamigni F., Rapezzi C., Ferrari R. (2021). Myocarditis in COVID-19 patients: Current problems. Intern. Emerg. Med..

[3] Bock C.-T., Klingel K., Kandolf R. (2010). Human Parvovirus B19–Associated Myocarditis. N. Engl. J. Med..

[4] Bowles N.E., Ni J., Kearney D.L., Pauschinger M., Schultheiss H.P., McCarthy R., Hare J., Bricker J.T., Bowles K.R., Towbin J.A. (2003). Detection of viruses in myocardial tissues by polymerase chain reaction: Evidence of adenovirus as a common cause of myocarditis in children and adults. J. Am. Coll. Cardiol..

[5] Lasrado N., Reddy J. (2020). An overview of the immune mechanisms of viral myocarditis. Rev. Med. Virol..

[6] Kühl U., Pauschinger M., Seeberg B., Lassner D., Noutsias M., Poller W., Schultheiss H.-P. (2005). Viral Persistence in the Myocardium Is Associated with Progressive Cardiac Dysfunction. Circulation.

[7] Kearney M., Cotton J., Richardson P., Shah A. (2001). Viral myocarditis and dilated cardiomyopathy: Mechanisms, manifestations, and management. Postgrad. Med. J..

[8] Reichart D., Magnussen C., Zeller T., Blankenberg S. (2019). Dilated cardiomyopathy: From epidemiologic to genetic phenotypes. J. Intern. Med..

[9] Weintraub R.G., Semsarian C., Macdonald P. (2017). Dilated cardiomyopathy. Lancet.

[10] Leonard E.G. (2004). Viral Myocarditis, Pediatr. Infect. Dis. J..

[11] Knowlton K.U. (2019). Dilated Cardiomyopathy. Circulation.

[12] Chau D.H.W., Yuan J., Zhang H., Cheung P., Lim T., Liu Z., Sall A., Yang D. (2007). Coxsackievirus B3 proteases 2A and 3C induce apoptotic cell death through mitochondrial injury and cleavage of eIF4GI but not DAP5/p97/NAT1. Apoptosis.

[13] Qiu Y., Ye X., Zhang H.M., Hanson P., Zhao G., Tong L., Xie R., Yang D. (2017). Cleavage of osmosensitive transcriptional factor NFAT5 by Coxsackieviral protease 2A promotes viral replication. PLoS Pathog..

[14] Zhao G., Zhang H.M., Qiu Y., Ye X., Yang D. (2020). Cleavage of Desmosomal Cadherins Promotes γ-Catenin Degradation and Benefits Wnt Signaling in Coxsackievirus B3-Induced Destruction of Cardiomyocytes. Front. Microbiol..

[15] Mohamud Y., Xue Y.C., Liu H., Ng C.S., Bahreyni A., Luo H. (2021). Autophagy Receptor Protein Tax1-Binding Protein 1/TRAF6-Binding Protein Is a Cellular Substrate of Enteroviral Proteinase. Front. Microbiol..

[16] Hanson P.J., Ye X., Qiu Y., Zhang H.M., Hemida M.G., Wang F., Lim T., Gu A., Cho B., Kim H. (2016). Cleavage of DAP5 by coxsackievirus B3 2A protease facilitates viral replication and enhances apoptosis by altering translation of IRES-containing genes. Cell Death Differ..

[17] Mukherjee A., Morosky S.A., Delorme-Axford E., Dybdahl-Sissoko N., Oberste M.S., Wang T., Coyne C.B. (2011). The Coxsackievirus B 3Cpro Protease Cleaves MAVS and TRIF to Attenuate Host Type I Interferon and Apoptotic Signaling. PLoS Pathog..

[18] Deonarain R., Cerullo D., Fuse K., Liu P.P., Fish E.N. (2004). Protective Role for Interferon-β in Coxsackievirus B3 Infection. Circulation.

[19] Chattopadhyay S., Marques J.T., Yamashita M., Peters K.L., Smith K., Desai A., Williams B.R., Sen G.C. (2010). Viral apoptosis is induced by IRF-3-mediated activation of Bax. EMBO J..

[20] Lei Y., Moore C.B., Liesman R.M., O’Connor B.P., Bergstralh D.T., Chen Z.J., Pickles R.J., Ting J.P.-Y. (2009). MAVS-Mediated Apoptosis and Its Inhibition by Viral Proteins. PLoS ONE.

[21] Kaiser W.J., Offermann M.K. (2005). Apoptosis Induced by the Toll-Like Receptor Adaptor TRIF Is Dependent on Its Receptor Interacting Protein Homotypic Interaction Motif. J. Immunol..

[22] Johnson Z.I., Doolittle A.C., Snuggs J.W., Shapiro I.M., Le Maitre C.L., Risbud M.V. (2017). TNF-α promotes nuclear enrichment of the transcription factor TonEBP/NFAT5 to selectively control inflammatory but not osmoregulatory responses in nucleus pulposus cells. J. Biol. Chem..

[23] Shi J., Wong J., Piesik P., Fung G., Zhang J., Jagdeo J., Li X., Jan E., Luo H. (2013). Cleavage of sequestosome 1/p62 by an enteroviral protease results in disrupted selective autophagy and impaired NFKB signaling. Autophagy.

[24] Rahnefeld A., Klingel K., Schuermann A., Diny N.L., Althof N., Lindner A., Bleienheuft P., Savvatis K., Respondek D., Opitz E. (2014). Ubiquitin-Like Protein ISG15 (Interferon-Stimulated Gene of 15 kDa) in Host Defense Against Heart Failure in a Mouse Model of Virus-Induced Cardiomyopathy. Circulation.

[25] Nadkarni R., Chu W.C., Lee C.Q.E., Mohamud Y., Yap L., Toh G.A., Beh S., Lim R., Fan Y.M., Zhang Y.L. (2022). Viral proteases activate the CARD8 inflammasome in the human cardiovascular system. J. Exp. Med..

[26] Gorbea C., Makar K.A., Pauschinger M., Pratt G., Bersola J.L.F., Varela J., David R.M., Banks L., Huang C.H., Li H. (2010). A Role for Toll-like Receptor 3 Variants in Host Susceptibility to Enteroviral Myocarditis and Dilated Cardiomyopathy. J. Biol. Chem..

[27] Alexopoulou L., Holt A.C., Medzhitov R., Flavell R.A. (2001). Recognition of double-stranded RNA and activation of NF-κB by Toll-like receptor 3. Nature.

[28] Jensen S., Thomsen A.R. (2012). Sensing of RNA Viruses: A Review of Innate Immune Receptors Involved in Recognizing RNA Virus Invasion. J. Virol..

[29] Triantafilou K., Orthopoulos G., Vakakis E., Ahmed M.A.E., Golenbock D.T., Lepper P.M., Triantafilou M. (2005). Human cardiac inflammatory responses triggered by Coxsackie B viruses are mainly Toll-like receptor (TLR) 8-dependent. Cell. Microbiol..

[30] Kumagai Y., Takeuchi O., Akira S. (2008). TLR9 as a key receptor for the recognition of DNA. Adv. Drug Deliv. Rev..

[31] Kawasaki T., Kawai T. (2014). Toll-Like Receptor Signaling Pathways. Front. Immunol..

[32] Kato H., Takeuchi O., Sato S., Yoneyama M., Yamamoto M., Matsui K., Uematsu S., Jung A., Kawai T., Ishii K.J. (2006). Differential roles of MDA5 and RIG-I helicases in the recognition of RNA viruses. Nature.

[33] Michael Lavigne G., Russell H., Sherry B., Ke R. (2021). Autocrine and paracrine interferon signalling as ‘ring vaccination’ and ‘contact tracing’ strategies to suppress virus infection in a host. Proc. R. Soc. B Biol. Sci..

[34] Smolgovsky S., Ibeh U., Tamayo T.P., Alcaide P. (2021). Adding insult to injury—Inflammation at the heart of cardiac fibrosis. Cell. Signal..

[35] Azevedo P.S., Polegato B.F., Minicucci M.F., Paiva S.A.R., Zornoff L.A.M. (2016). Cardiac Remodeling: Concepts, Clinical Impact, Pathophysiological Mechanisms and Pharmacologic Treatment. Arq. Bras. Cardiol..

[36] Xu D., Wang P., Yang J., Qian Q., Li M., Wei L., Xu W. (2018). Gr-1+ Cells Other Than Ly6G+ Neutrophils Limit Virus Replication and Promote Myocardial Inflammation and Fibrosis Following Coxsackievirus B3 Infection of Mice. Front. Cell. Infect. Microbiol..

[37] Hammond M.D., Ai Y., Sansing L.H. (2012). Gr1+ Macrophages and Dendritic Cells Dominate the Inflammatory Infiltrate 12 h After Experimental Intracerebral Hemorrhage. Transl. Stroke Res..

[38] Niedermeier M., Reich B., Gomez M.R., Denzel A., Schmidbauer K., Göbel N., Talke Y., Schweda F., Mack M. (2009). CD4+ T cells control the differentiation of Gr1+ monocytes into fibrocytes. Proc. Natl. Acad. Sci. USA.

[39] Beinert H., Holm R.H., Münck E. (1997). Iron-Sulfur Clusters: Nature’s Modular, Multipurpose Structures. Science.

[40] Naito Y., Tsujino T., Matsumoto M., Sakoda T., Ohyanagi M., Masuyama T. (2009). Adaptive response of the heart to long-term anemia induced by iron deficiency. Am. J. Physiol. Heart Circ. Physiol..

[41] Hoes M.F., Grote Beverborg N., Kijlstra J.D., Kuipers J., Swinkels D.W., Giepmans B.N.G., Rodenburg R.J., van Veldhuisen D.J., de Boer R.A., van der Meer P. (2018). Iron deficiency impairs contractility of human cardiomyocytes through decreased mitochondrial function. Eur. J. Heart Fail..

[42] Kobak K.A., Franczuk P., Schubert J., Dzięgała M., Kasztura M., Tkaczyszyn M., Drozd M., Kosiorek A., Kiczak L., Bania J. (2021). Primary Human Cardiomyocytes and Cardiofibroblasts Treated with Sera from Myocarditis Patients Exhibit an Increased Iron Demand and Complex Changes in the Gene Expression. Cells.

[43] Pelosi E., Testa U., Louache F., Thomopoulos P., Salvo G., Samoggia P., Peschle C. (1986). Expression of transferrin receptors in phytohemagglutinin-stimulated human T-lymphocytes. Evidence for a three-step model. J. Biol. Chem..

[44] Lanser L., Nemati N., Seifert M., Fuchs D., Weiss G., Pölzl G., Kurz K. (2020). Inflammation, iron and vitamin D metabolism in different cardiomyopathy aetiologies. Pteridines.

[45] Li J., Cao F., Yin H., Huang Z., Lin Z., Mao N., Sun B., Wang G. (2020). Ferroptosis: Past, present and future. Cell Death Dis..

[46] Yi L., Hu Y., Wu Z., Li Y., Kong M., Kang Z., Zuoyuan B., Yang Z. (2022). TFRC upregulation promotes ferroptosis in CVB3 infection via nucleus recruitment of Sp1. Cell Death Dis..

[47] Hong M., Rong J., Tao X., Xu Y. (2022). The Emerging Role of Ferroptosis in Cardiovascular Diseases. Front. Pharmacol..

[48] Ursu O.N., Sauter M., Ettischer N., Kandolf R., Klingel K. (2014). Heme Oxygenase-1 Mediates Oxidative Stress and Apoptosis in Coxsackievirus B3-Induced Myocarditis. Cell. Physiol. Biochem..

[49] Liu X., Li M., Chen Z., Yu Y., Shi H., Yu Y., Wang Y., Chen R., Ge J. (2022). Mitochondrial calpain-1 activates NLRP3 inflammasome by cleaving ATP5A1 and inducing mitochondrial ROS in CVB3-induced myocarditis. Basic Res. Cardiol..

[50] Shi H., Yu Y., Liu X., Yu Y., Li M., Wang Y., Zou Y., Chen R., Ge J. (2022). Inhibition of calpain reduces cell apoptosis by suppressing mitochondrial fission in acute viral myocarditis. Cell Biol. Toxicol..

[51] Oh S.-J., Lim B.-K., Yun J., Shin O.S. (2021). CVB3-Mediated Mitophagy Plays an Important Role in Viral Replication via Abrogation of Interferon Pathways. Front. Cell. Infect. Microbiol..

[52] Sin J., McIntyre L., Stotland A., Feuer R., Gottlieb R.A. (2017). Coxsackievirus B Escapes the Infected Cell in Ejected Mitophagosomes. J. Virol..

[53] Bu L., Wang H., Hou P., Guo S., He M., Xiao J., Li P., Zhong Y., Jia P., Cao Y. (2020). The Ubiquitin E3 Ligase Parkin Inhibits Innate Antiviral Immunity Through K48-Linked Polyubiquitination of RIG-I and MDA5. Front. Immunol..

[54] Remels A.H.V., Derks W.J.A., Cillero-Pastor B., Verhees K.J.P., Kelders M.C., Heggermont W., Carai P., Summer G., Ellis S.R., de Theije C.C. (2018). NF-κB-mediated metabolic remodelling in the inflamed heart in acute viral myocarditis. Biochim. Biophys. Acta BBA Mol. Basis Dis..

[55] Fisher R.P., Clayton D.A. (1988). Purification and characterization of human mitochondrial transcription factor 1. Mol. Cell. Biol..

[56] Larsson N.-G., Wang J., Wilhelmsson H., Oldfors A., Rustin P., Lewandoski M., Barsh G.S., Clayton D.A. (1998). Mitochondrial transcription factor A is necessary for mtDNA maintance and embryogenesis in mice. Nat. Genet..

[57] LeBleu V.S., O’Connell J.T., Herrera K.N.G., Wikman-Kocher H., Pantel K., Haigis M.C., de Carvalho F.M., Damascena A., Chinen L.T.D., Rocha R.M. (2014). PGC-1α mediates mitochondrial biogenesis and oxidative phosphorylation to promote metastasis. Nat. Cell Biol..

[58] Dassanayaka S., Jones S.P. (2014). O-GlcNAc and the cardiovascular system. Pharmacol. Ther..

[59] Paradies G., Paradies V., Ruggiero F.M., Petrosillo G. (2014). Oxidative stress, cardiolipin and mitochondrial dysfunction in nonalcoholic fatty liver disease. World J. Gastroenterol. WJG.

[60] Davies P., Bailey P.J., Goldenberg M.M., Ford-Hutchinson A.W. (1984). The Role of Arachidonic Acid Oxygenation Products in Pain and Inflammation. Annu. Rev. Immunol..

[61] Fang H., Judd R.L. (2018). Adiponectin regulation and function. Comprehensive Physiology.

[62] Skurk C., Wittchen F., Suckau L., Witt H., Noutsias M., Fechner H., Schultheiss H.-P., Poller W. (2008). Description of a local cardiac adiponectin system and its deregulation in dilated cardiomyopathy. Eur. Heart J..

[63] Jenke A., Holzhauser L., Löbel M., Savvatis K., Wilk S., Weithäuser A., Pinkert S., Tschöpe C., Klingel K., Poller W. (2014). Adiponectin promotes coxsackievirus B3 myocarditis by suppression of acute anti-viral immune responses. Basic Res. Cardiol..

[64] Frisancho-Kiss S., Nyland J.F., Davis S.E., Augusto Frisancho J., Barrett M.A., Rose N.R., Fairweather D. (2006). Sex differences in coxsackievirus B3-induced myocarditis: IL-12Rβ1 signaling and IFN-γ increase inflammation in males independent from STAT4. Brain Res..

[65] Fairweather D., Yusung S., Frisancho S., Barrett M., Gatewood S., Steele R., Rose N.R. (2003). IL-12 Receptor β1 and Toll-Like Receptor 4 Increase IL-1β- and IL-18-Associated Myocarditis and Coxsackievirus Replication. J. Immunol..

[66] Cihakova D., Barin J.G., Afanasyeva M., Kimura M., Fairweather D., Berg M., Talor M.V., Baldeviano G.C., Frisancho S., Gabrielson K. (2008). Interleukin-13 Protects Against Experimental Autoimmune Myocarditis by Regulating Macrophage Differentiation. Am. J. Pathol..

[67] Choi J.-M., Bothwell A.L.M. (2012). The Nuclear Receptor PPARs as Important Regulators of T-Cell Functions and Autoimmune Diseases. Mol. Cells.

[68] Chang H., Zhao F., Xie X., Liao Y., Song Y., Liu C., Wu Y., Wang Y., Liu D., Wang Y. (2019). PPARα suppresses Th17 cell differentiation through IL-6/STAT3/RORγt pathway in experimental autoimmune myocarditis. Exp. Cell Res..

[69] Zhou L., Lopes J.E., Chong M.M.W., Ivanov I.I., Min R., Victora G.D., Shen Y., Du J., Rubtsov Y.P., Rudensky A.Y. (2008). TGF-β-induced Foxp3 inhibits Th17 cell differentiation by antagonizing RORγt function. Nature.

[70] Kersten S., Desvergne B., Wahli W. (2000). Roles of PPARs in health and disease. Nature.

[71] Tan H.W.S., Anjum B., Shen H.-M., Ghosh S., Yen P.M., Sinha R.A. (2019). Lysosomal inhibition attenuates peroxisomal gene transcription via suppression of PPARA and PPARGC1A levels. Autophagy.

[72] Handschin C., Spiegelman B.M. (2006). Peroxisome Proliferator-Activated Receptor γ Coactivator 1 Coactivators, Energy Homeostasis, and Metabolism. Endocr. Rev..

[73] Riehle C., Wende A.R., Zaha V.G., Pires K.M., Wayment B., Olsen C., Bugger H., Buchanan J., Wang X., Moreira A.B. (2011). PGC-1β Deficiency Accelerates the Transition to Heart Failure in Pressure Overload Hypertrophy. Circ. Res..

[74] Arany Z., Novikov M., Chin S., Ma Y., Rosenzweig A., Spiegelman B.M. (2006). Transverse aortic constriction leads to accelerated heart failure in mice lacking PPAR-γ coactivator 1α. Proc. Natl. Acad. Sci. USA.

[75] Yang J., Zhang L., Yu C., Yang X.-F., Wang H. (2014). Monocyte and macrophage differentiation: Circulation inflammatory monocyte as biomarker for inflammatory diseases. Biomark. Res..

[76] Golubovskaya V., Wu L. (2016). Different Subsets of T Cells, Memory, Effector Functions, and CAR-T Immunotherapy. Cancers.

[77] Jackson S.M., Wilson P.C., James J.A., Capra J.D. (2008). Human B cell subsets. Advances in Immunology.

[78] MacKenna D., Summerour S.R., Villarreal F.J. (2000). Role of mechanical factors in modulating cardiac fibroblast function and extracellular matrix synthesis. Cardiovasc. Res..

[79] Yang X., Yue Y., Xiong S. (2019). Dpep2 Emerging as a Modulator of Macrophage Inflammation Confers Protection Against CVB3-Induced Viral Myocarditis. Front. Cell. Infect. Microbiol..

[80] Carai P., Ruozi G., Paye A., Debing Y., Bortolotti F., Lecomte J., Zentilin L., Jones E.A.V., Giacca M., Heymans S. (2022). AAV9-mediated functional screening for cardioprotective cytokines in Coxsackievirus-B3-induced myocarditis. Sci. Rep..

[81] Yu M., Long Q., Li H.-H., Liang W., Liao Y.-H., Yuan J., Cheng X. (2016). IL-9 Inhibits Viral Replication in Coxsackievirus B3-Induced Myocarditis. Front. Immunol..

[82] Dhingra S., Sharma A.K., Arora R.C., Slezak J., Singal P.K. (2009). IL-10 attenuates TNF-α-induced NFκB pathway activation and cardiomyocyte apoptosis. Cardiovasc. Res..

[83] Harris S.G., Padilla J., Koumas L., Ray D., Phipps R.P. (2002). Prostaglandins as modulators of immunity. Trends Immunol..

[84] YlÖstalo J.H., Bartosh T.J., Coble K., Prockop D.J. (2012). Human Mesenchymal Stem/Stromal Cells Cultured as Spheroids are Self-activated to Produce Prostaglandin E2 that Directs Stimulated Macrophages into an Anti-inflammatory Phenotype. Stem Cells..

[85] Loynes C.A., Lee J.A., Robertson A.L., Steel M.J.G., Ellett F., Feng Y., Levy B.D., Whyte M.K.B., Renshaw S.A. (2018). PGE2 production at sites of tissue injury promotes an anti-inflammatory neutrophil phenotype and determines the outcome of inflammation resolution in vivo. Sci. Adv..

[86] Chen R., Cao Y., Tian Y., Gu Y., Lu H., Zhang S., Xu H., Su Z. (2020). PGE2 ameliorated viral myocarditis development and promoted IL-10-producing regulatory B cell expansion via MAPKs/AKT-AP1 axis or AhR signaling. Cell. Immunol..

[87] Li L., Li L., Xiao L., Shangguan J. (2018). Progranulin ameliorates coxsackievirus-B3-induced viral myocarditis by downregulating Th1 and Th17 cells. Exp. Cell Res..

[88] Carai P., Papageorgiou A.P., Van Linthout S., Deckx S., Velthuis S., Lutgens E., Wijnands E., Tschöpe C., Schmuttermaier C., Kzhyshkowska J. (2022). Stabilin-1 mediates beneficial monocyte recruitment and tolerogenic macrophage programming during CVB3-induced viral myocarditis. J. Mol. Cell. Cardiol..

[89] Kratofil R.M., Kubes P., Deniset J.F. (2017). Monocyte Conversion During Inflammation and Injury. Arterioscler. Thromb. Vasc. Biol..

[90] Leuschner F., Courties G., Dutta P., Mortensen L.J., Gorbatov R., Sena B., Novobrantseva T.I., Borodovsky A., Fitzgerald K., Koteliansky V. (2015). Silencing of CCR2 in myocarditis. Eur. Heart J..

[91] Tsou C.-L., Peters W., Si Y., Slaymaker S., Aslanian A.M., Weisberg S.P., Mack M., Charo I.F. (2007). Critical roles for CCR2 and MCP-3 in monocyte mobilization from bone marrow and recruitment to inflammatory sites. J. Clin. Investig..

[92] Huang Y., Li Y., Wei B., Wu W., Gao X. (2018). CD80 Regulates Th17 Cell Differentiation in Coxsackie Virus B3-Induced Acute Myocarditis. Inflammation.

[93] De Giusti C.J., Ure A.E., Rivadeneyra L., Schattner M., Gomez R.M. (2015). Macrophages and galectin 3 play critical roles in CVB3-induced murine acute myocarditis and chronic fibrosis. J. Mol. Cell. Cardiol..

[94] Myers J.M., Cooper L.T., Kem D.C., Stavrakis S., Kosanke S.D., Shevach E.M., Fairweather D., Stoner J.A., Cox C.J., Cunningham M.W. (2016). Cardiac myosin-Th17 responses promote heart failure in human myocarditis. JCI Insight.

[95] McGeachy M.J. (2011). GM-CSF: The secret weapon in the TH17 arsenal. Nat. Immunol..

[96] Baldeviano G.C., Barin J.G., Talor M.V., Srinivasan S., Bedja D., Zheng D., Gabrielson K., Iwakura Y., Rose N.R., Cihakova D. (2010). Interleukin-17A Is Dispensable for Myocarditis but Essential for the Progression to Dilated Cardiomyopathy. Circ. Res..

[97] Kraft L., Erdenesukh T., Sauter M., Tschöpe C., Klingel K. (2019). Blocking the IL-1β signalling pathway prevents chronic viral myocarditis and cardiac remodeling. Basic Res. Cardiol..

[98] Al-Kofahi M., Omura S., Tsunoda I., Sato F., Becker F., Gavins F.N., Woolard M.D., Pattillo C., Zawieja D., Muthuchamy M. (2018). IL-1β reduces cardiac lymphatic muscle contraction via COX-2 and PGE2 induction: Potential role in myocarditis. Biomed. Pharmacother..

[99] Savvatis K., Müller I., Fröhlich M., Pappritz K., Zietsch C., Hamdani N., Grote K., Schieffer B., Klingel K., Van Linthout S. (2014). Interleukin-6 receptor inhibition modulates the immune reaction and restores titin phosphorylation in experimental myocarditis. Basic Res. Cardiol..

[100] Valaperti A., Nishii M., Liu Y., Naito K., Chan M., Zhang L., Skurk C., Schultheiss H.-P., Wells G.A., Eriksson U. (2013). Innate Immune Interleukin-1 Receptor–Associated Kinase 4 Exacerbates Viral Myocarditis by Reducing CCR5+CD11b+ Monocyte Migration and Impairing Interferon Production. Circulation.

[101] López B., González A., Lindner D., Westermann D., Ravassa S., Beaumont J., Gallego I., Zudaire A., Brugnolaro C., Querejeta R. (2013). Osteopontin-mediated myocardial fibrosis in heart failure: A role for lysyl oxidase?. Cardiovasc. Res..

[102] Antoniak S., Owens A.P., Baunacke M., Williams J.C., Lee R.D., Weithäuser A., Sheridan P.A., Malz R., Luyendyk J.P., Esserman D.A. (2013). PAR-1 contributes to the innate immune response during viral infection. J. Clin. Investig..

[103] Weithauser A., Bobbert P., Antoniak S., Böhm A., Rauch B.H., Klingel K., Savvatis K., Kroemer H.K., Tschope C., Stroux A. (2013). Protease-Activated Receptor-2 Regulates the Innate Immune Response to Viral Infection in a Coxsackievirus B3–Induced Myocarditis. J. Am. Coll. Cardiol..

[104] Müller I., Vogl T., Pappritz K., Miteva K., Savvatis K., Rohde D., Most P., Lassner D., Pieske B., Kühl U. (2017). Pathogenic Role of the Damage-Associated Molecular Patterns S100A8 and S100A9 in Coxsackievirus B3–Induced Myocarditis. Circ. Heart Fail..

[105] Li Y., Chen B., Yang X., Zhang C., Jiao Y., Li P., Liu Y., Li Z., Qiao B., Bond Lau W. (2019). S100a8/a9 Signaling Causes Mitochondrial Dysfunction and Cardiomyocyte Death in Response to Ischemic/Reperfusion Injury. Circulation.

[106] Xue Y.-L., Zhang S.-X., Zheng C.-F., Li Y.-F., Zhang L.-H., Hao Y.-F., Wang S., Li X.-W. (2019). Silencing of STAT4 Protects Against Autoimmune Myocarditis by Regulating Th1/Th2 Immune Response via Inactivation of the NF-κB Pathway in Rats. Inflammation.

[107] Bhattacharyya S., Wang W., Morales-Nebreda L., Feng G., Wu M., Zhou X., Lafyatis R., Lee J., Hinchcliff M., Feghali-Bostwick C. (2016). Tenascin-C drives persistence of organ fibrosis. Nat. Commun..

[108] Juni R.P., Kuster D.W.D., Goebel M., Helmes M., Musters R.J.P., van der Velden J., Koolwijk P., Paulus W.J., van Hinsbergh V.W.M. (2019). Cardiac Microvascular Endothelial Enhancement of Cardiomyocyte Function Is Impaired by Inflammation and Restored by Empagliflozin. JACC Basic Transl. Sci..

[109] Sun M., Chen M., Dawood F., Zurawska U., Li J.Y., Parker T., Kassiri Z., Kirshenbaum L.A., Arnold M., Khokha R. (2007). Tumor Necrosis Factor-α Mediates Cardiac Remodeling and Ventricular Dysfunction After Pressure Overload State. Circulation.

[110] Huber S.A., Sartini D. (2005). Roles of Tumor Necrosis Factor Alpha (TNF-α) and the p55 TNF Receptor in CD1d Induction and Coxsackievirus B3-Induced Myocarditis. J. Virol..

[111] Suematsu N., Tsutsui H., Wen J., Kang D., Ikeuchi M., Ide T., Hayashidani S., Shiomi T., Kubota T., Hamasaki N. (2003). Oxidative Stress Mediates Tumor Necrosis Factor-α–Induced Mitochondrial DNA Damage and Dysfunction in Cardiac Myocytes. Circulation.

[112] Fang M., Zhang A., Du Y., Lu W., Wang J., Minze L.J., Cox T.C., Li X.C., Xing J., Zhang Z. (2022). TRIM18 is a critical regulator of viral myocarditis and organ inflammation. J. Biomed. Sci..

[113] Lasrado N., Borcherding N., Arumugam R., Starr T.K., Reddy J. (2022). Dissecting the cellular landscape and transcriptome network in viral myocarditis by single-cell RNA sequencing. IScience.

[114] Barnes B.J., Field A.E., Pitha-Rowe P.M. (2003). Virus-induced Heterodimer Formation betweenIRF-5 and IRF-7 Modulates Assembly of theIFNA Enhanceosome in Vivo and Transcriptional Activity of IFNA Genes. J. Biol. Chem..

[115] Garlanda C., Dinarello C.A., Mantovani A. (2013). The Interleukin-1 Family: Back to the Future. Immunity.

[116] Dinarello C.A. (2011). Interleukin-1 in the pathogenesis and treatment of inflammatory diseases. Blood.

[117] Henderson N.C., Mackinnon A.C., Farnworth S.L., Poirier F., Russo F.P., Iredale J.P., Haslett C., Simpson K.J., Sethi T. (2006). Galectin-3 regulates myofibroblast activation and hepatic fibrosis. Proc. Natl. Acad. Sci. USA.

[118] Sharma U., Rhaleb N.-E., Pokharel S., Harding P., Rasoul S., Peng H., Carretero O.A. (2008). Novel anti-inflammatory mechanisms of N-Acetyl-Ser-Asp-Lys-Pro in hypertension-induced target organ damage. Am. J. Physiol. Heart Circ. Physiol..

[119] Yang D., Han Z., Oppenheim J.J. (2017). Alarmins and immunity. Immunol. Rev..

[120] Rider P., Voronov E., Dinarello C.A., Apte R.N., Cohen I. (2017). Alarmins: Feel the Stress. J. Immunol..

[121] Wang S., Song R., Wang Z., Jing Z., Wang S., Ma J. (2018). S100A8/A9 in Inflammation. Front. Immunol..

[122] Averill M.M., Kerkhoff C., Bornfeldt K.E. (2012). S100A8 and S100A9 in Cardiovascular Biology and Disease. Arterioscler. Thromb. Vasc. Biol..

[123] Yang J., Anholts J., Kolbe U., Stegehuis-Kamp J.A., Claas F.H.J., Eikmans M. (2018). Calcium-Binding Proteins S100A8 and S100A9: Investigation of Their Immune Regulatory Effect in Myeloid Cells. Int. J. Mol. Sci..

[124] Crowe L.A.N., McLean M., Kitson S.M., Melchor E.G., Patommel K., Cao H.M., Reilly J.H., Leach W.J., Rooney B.P., Spencer S.J. (2019). S100A8 & S100A9: Alarmin mediated inflammation in tendinopathy. Sci. Rep..

[125] Guo Q., Zhao Y., Li J., Liu J., Yang X., Guo X., Kuang M., Xia H., Zhang Z., Cao L. (2021). Induction of alarmin S100A8/A9 mediates activation of aberrant neutrophils in the pathogenesis of COVID-19. Cell Host Microbe.

[126] Szalay G., Sauter M., Hald J., Weinzierl A., Kandolf R., Klingel K. (2006). Sustained Nitric Oxide Synthesis Contributes to Immunopathology in Ongoing Myocarditis Attributable to Interleukin-10 Disorders. Am. J. Pathol..

[127] Sreejit G., Abdel-Latif A., Athmanathan B., Annabathula R., Dhyani A., Noothi S.K., Quaife-Ryan G.A., Al-Sharea A., Pernes G., Dragoljevic D. (2020). Neutrophil-Derived S100A8/A9 Amplify Granulopoiesis After Myocardial Infarction. Circulation.

[128] Haller O., Stertz S., Kochs G. (2007). The Mx GTPase family of interferon-induced antiviral proteins. Microbes Infect..

[129] Hwang S.-M., Sharma G., Verma R., Byun S., Rudra D., Im S.-H. (2018). Inflammation-induced Id2 promotes plasticity in regulatory T cells. Nat. Commun..

[130] Gassner F.J., Zaborsky N., Catakovic K., Rebhandl S., Huemer M., Egle A., Hartmann T.N., Greil R., Geisberger R. (2015). Chronic lymphocytic leukaemia induces an exhausted T cell phenotype in the TCL1 transgenic mouse model. Br. J. Haematol..

[131] Ng S.S., De Labastida Rivera F., Yan J., Corvino D., Das I., Zhang P., Kuns R., Chauhan S.B., Hou J., Li X.-Y. (2020). The NK cell granule protein NKG7 regulates cytotoxic granule exocytosis and inflammation. Nat. Immunol..

[132] Basavalingappa R.H., Arumugam R., Lasrado N., Yalaka B., Massilamany C., Gangaplara A., Riethoven J.-J., Xiang S.-H., Steffen D., Reddy J. (2020). Viral myocarditis involves the generation of autoreactive T cells with multiple antigen specificities that localize in lymphoid and non-lymphoid organs in the mouse model of CVB3 infection. Mol. Immunol..

[133] Bunte K., Beikler T. (2019). Th17 Cells and the IL-23/IL-17 Axis in the Pathogenesis of Periodontitis and Immune-Mediated Inflammatory Diseases. Int. J. Mol. Sci..

[134] Kimura A., Kishimoto T. (2010). IL-6: Regulator of Treg/Th17 balance. Eur. J. Immunol..

[135] Kara E.E., McKenzie D.R., Bastow C.R., Gregor C.E., Fenix K.A., Ogunniyi A.D., Paton J.C., Mack M., Pombal D.R., Seillet C. (2015). CCR2 defines in vivo development and homing of IL-23-driven GM-CSF-producing Th17 cells. Nat. Commun..

[136] Deswal A., Petersen N.J., Feldman A.M., Young J.B., White B.G., Mann D.L. (2001). Cytokines and Cytokine Receptors in Advanced Heart Failure. Circulation.

[137] Rudd C.E., Taylor A., Schneider H. (2009). CD28 and CTLA-4 coreceptor expression and signal transduction. Immunol. Rev..

[138] Korn T., Bettelli E., Oukka M., Kuchroo V.K. (2009). IL-17 and Th17 Cells. Annu. Rev. Immunol..

[139] Satoh M., Nakamura M., Saitoh H., Satoh H., Maesawa C., Segawa I., Tashiro A., Hiramori K. (1999). Tumor Necrosis Factor-α–Converting Enzyme and Tumor Necrosis Factor-α in Human Dilated Cardiomyopathy. Circulation.

[140] Levine B., Kalman J., Mayer L., Fillit H.M., Packer M. (1990). Elevated Circulating Levels of Tumor Necrosis Factor in Severe Chronic Heart Failure. N. Engl. J. Med..

[141] Medeiros N.I., Gomes J.A.S., Fiuza J.A., Sousa G.R., Almeida E.F., Novaes R.O., Rocha V.L.S., Chaves A.T., Dutra W.O., Rocha M.O.C. (2019). MMP-2 and MMP-9 plasma levels are potential biomarkers for indeterminate and cardiac clinical forms progression in chronic Chagas disease. Sci. Rep..

[142] Westermann D., Savvatis K., Schultheiss H.P., Tschöpe C. (2010). Immunomodulation and matrix metalloproteinases in viral myocarditis. J. Mol. Cell. Cardiol..

[143] Cheung C., Marchant D., Walker E.K.-Y., Luo Z., Zhang J., Yanagawa B., Rahmani M., Cox J., Overall C., Senior R.M. (2008). Ablation of Matrix Metalloproteinase-9 Increases Severity of Viral Myocarditis in Mice. Circulation.

[144] Diny N.L., Hou X., Barin J.G., Chen G., Talor M.V., Schaub J., Russell S.D., Klingel K., Rose N.R., Čiháková D. (2016). Macrophages and cardiac fibroblasts are the main producers of eotaxins and regulate eosinophil trafficking to the heart. Eur. J. Immunol..

[145] Daniel R., He Z., Carmichael K.P., Halper J., Bateman A. (2000). Cellular Localization of Gene Expression for Progranulin. J. Histochem. Cytochem..

[146] Liu W., Dienz O., Roberts B., Moussawi M., Rincon M., Huber S.A. (2012). IL-21R expression on CD8^+^ T cells promotes CD8^+^ T cell activation in coxsackievirus B3 induced myocarditis. Exp. Mol. Pathol..

[147] Adamo L., Rocha-Resende C., Lin C.-Y., Evans S., Williams J., Dun H., Li W., Mpoy C., Andhey P.S., Rogers B.E. (2020). Myocardial B cells are a subset of circulating lymphocytes with delayed transit through the heart. JCI Insight.

[148] Cen Z., Li Y., Wei B., Wu W., Huang Y., Lu J. (2021). The Role of B Cells in Regulation of Th Cell Differentiation in Coxsackievirus B3–Induced Acute Myocarditis. Inflammation.

[149] Wei B., Deng Y., Huang Y., Gao X., Wu W. (2019). IL-10-producing B cells attenuate cardiac inflammation by regulating Th1 and Th17 cells in acute viral myocarditis induced by coxsackie virus B3. Life Sci..

[150] Yu M., Wen S., Wang M., Liang W., Li H.-H., Long Q., Guo H.P., Liao Y.-H., Yuan J. (2013). TNF-α-Secreting B Cells Contribute to Myocardial Fibrosis in Dilated Cardiomyopathy. J. Clin. Immunol..

[151] Stoian I., Oros A., Moldoveanu E. (1996). Apoptosis and Free Radicals. Biochem. Mol. Med..

[152] Tsutamoto T., Wada A., Matsumoto T., Maeda K., Mabuchi N., Hayashi M., Tsutsui T., Ohnishi M., Sawaki M., Fujii M. (2001). Relationship between tumor necrosis factor-alpha production and oxidative stress in the failing hearts of patients with dilated cardiomyopathy. J. Am. Coll. Cardiol..

[153] Luo Y., Zhang H., Yu J., Wei L., Li M., Xu W. (2022). Stem cell factor/mast cell/CCL2/monocyte/macrophage axis promotes Coxsackievirus B3 myocarditis and cardiac fibrosis by increasing Ly6Chigh monocyte influx and fibrogenic mediators production. Immunology.

[154] Hamdani N., Herwig M., Linke W.A. (2017). Tampering with springs: Phosphorylation of titin affecting the mechanical function of cardiomyocytes. Biophys. Rev..

[155] Kötter S., Kazmierowska M., Andresen C., Bottermann K., Grandoch M., Gorressen S., Heinen A., Moll J.M., Scheller J., Gödecke A. (2016). Titin-Based Cardiac Myocyte Stiffening Contributes to Early Adaptive Ventricular Remodeling After Myocardial Infarction. Circ. Res..

[156] Kötter S., Gout L., Von Frieling-Salewsky M., Müller A.E., Helling S., Marcus K., Dos Remedios C., Linke W.A., Krüger M. (2013). Differential changes in titin domain phosphorylation increase myofilament stiffness in failing human hearts. Cardiovasc. Res..

[157] Tanaka T., Narazaki M., Kishimoto T. (2012). Therapeutic Targeting of the Interleukin-6 Receptor. Annu. Rev. Pharmacol. Toxicol..

[158] Sébert M., Sola-Tapias N., Mas E., Barreau F., Ferrand A. (2019). Protease-Activated Receptors in the Intestine: Focus on Inflammation and Cancer. Front. Endocrinol..

[159] Heuberger D.M., Schuepbach R.A. (2019). Protease-activated receptors (PARs): Mechanisms of action and potential therapeutic modulators in PAR-driven inflammatory diseases. Thromb. J..

[160] Yuan J., Liu Z., Lim T., Zhang H., He J., Walker E., Shier C., Wang Y., Su Y., Sall A. (2009). CXCL10 Inhibits Viral Replication Through Recruitment of Natural Killer Cells in Coxsackievirus B3-Induced Myocarditis. Circ. Res..

[161] Bode M.F., Schmedes C.M., Egnatz G.J., Bharathi V., Hisada Y.M., Martinez D., Kawano T., Weithauser A., Rosenfeldt L., Rauch U. (2021). Cell type-specific roles of PAR1 in Coxsackievirus B3 infection. Sci. Rep..

[162] Mohamud Y., Shi J., Qu J., Poon T., Xue Y.C., Deng H., Zhang J., Luo H. (2018). Enteroviral Infection Inhibits Autophagic Flux via Disruption of the SNARE Complex to Enhance Viral Replication. Cell Rep..

